# Diffuse Multifocal Intracranial Hemorrhage Following Alteplase Infusion: A Case Image Report

**DOI:** 10.1002/ccr3.70263

**Published:** 2025-05-05

**Authors:** Abdallah Alwali, Mohanad Faisal, Naveed Akhtar

**Affiliations:** ^1^ Hamad Medical Corporation Doha Qatar

**Keywords:** Alteplas, hemorrhagic transformation, intracranial hemorrhage, thrombolysis

## Abstract

Hemorrhage beyond the ischemic area is an uncommon but serious complication of thrombolysis. This case underscores the importance of timely recognition of atypical bleeding sites, careful monitoring, and strict blood pressure control to improve outcomes in ischemic stroke patients post‐thrombolysis.

## 1 Case Presentation

A 70‐year‐old Sudanese male patient presented to the emergency department with acute‐onset left‐sided weakness and confusion, which began two hours before arriving at the emergency department. His medical background includes uncontrolled hypertension, diabetes mellitus, dyslipidaemia, and coronary artery disease. He underwent coronary artery bypass graft surgery 20 years ago but was not regularly taking antiplatelet medication. On presentation, blood pressure was 196/107 mmHg; a neurological exam revealed left side weakness (motor power 4/5 for both upper and lower limbs, proximally and distally), facial weakness with right gaze preference, and the patient was not able to follow commands. National Institutes of Health Stroke Scale (NIHSS) was 11, the Glasgow Coma Scale (GCS) was 13, and SEDAN Score was 3 points with an 8.8% risk of symptomatic intracranial hemorrhage (ICH).

Laboratory test results were significant for a high HbA1c level of 7.5%, borderline hypercholesterolemia, and elevated blood glucose (180 mg/dL). Hemoglobin and platelet counts were within normal limits; the prothrombin time (PT) was not checked either before or after thrombolysis. A computed tomography (CT) scan with CT perfusion revealed features suggestive of acute ischemic changes in the right temporoparietal area, with a surrounding penumbra. Magnetic Resonance Imaging (MRI) was not performed to rule out amyloid angiopathy.

**FIGURE 1:**
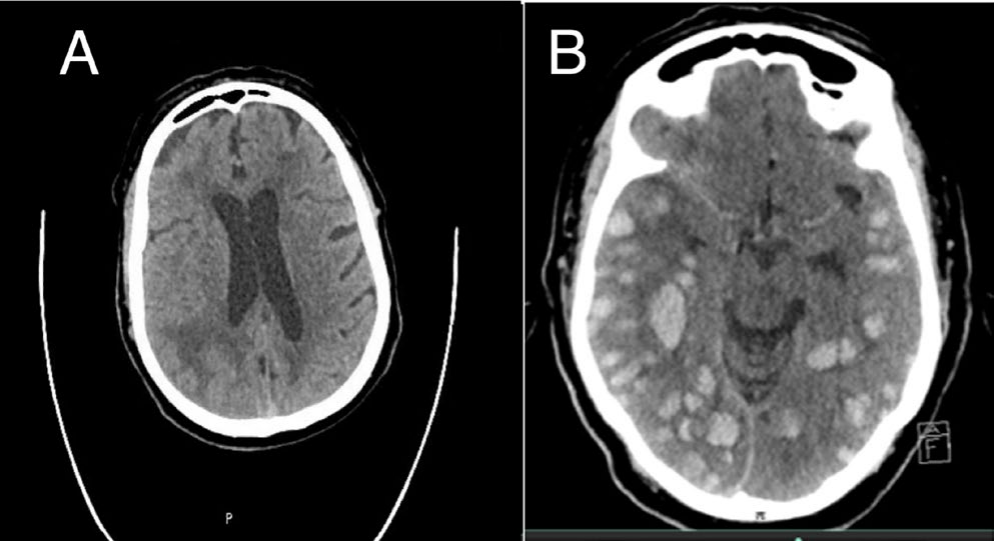
(A) Head CT scan showing hypo‐density in the right temporoparietal area. (B) Post‐thrombolysis head CT scan showing multifocal diffuse intracranial hemorrhage.

The patient received Alteplase, a tissue plasminogen activator (tPA), as a 90 mg infusion over 60 min following initial blood pressure management. Systolic blood pressure after thrombolysis fluctuated between 150 and 176 mmHg, reaching a peak of 197 mmHg, which was repeated after 10 min and subsequently decreased to 176 mmHg. However, after 6 h, the patient became suddenly less responsive, and the GCS dropped to 6. The urgent repeat CT scan revealed diffuse, numerous intra‐parenchymal bleeds, along with mild compression of the right lateral ventricle and midline shift (see Figure 1). Unfortunately, the patient's neurological condition was assessed regularly by stroke team, but it remained unchanged during his admission for 2 months. During the hospital course, despite receiving proper nursing and medical care, the patient experienced spikes of fever and developed hospital‐acquired pneumonia. This condition subsequently progressed to severe sepsis with multi‐organ dysfunction, ultimately leading to the patient's death.

## 2 Discussion

Haemorrhagic transformation of ischemic stroke refers to the secondary conversion of an ischemic infarct into an area of hemorrhage. It is considered a major limitation of intravenous thrombolysis using tPA, which can complicate the management and prognosis of ischemic stroke. The incidence of symptomatic intracranial hemorrhage (based on radiological classification and neurological status) generally ranges from 2% to 7% [1]. Risk factors associated with an increased risk of hemorrhagic transformation include older age, greater stroke severity, higher baseline glucose, uncontrolled blood pressures during and post‐thrombolysis, congestive heart failure, renal impairment, diabetes mellitus, ischemic heart disease, atrial fibrillation, baseline antiplatelet use, leucocytosis, and visible acute infarction on brain imaging [2]. The occurrence of ICH post‐tPA at a distant site from the ischemic stroke site is unusual, and specific risk factors are more closely associated with extraischemic intracerebral hemorrhage, such as a history of previous strokes and advanced age [3].

In this report, we highlight a multifocal hemorrhagic transformation beyond the primary site of the ischemic area as an important and unusual complication of thrombolysis therapy. We emphasize the need for close monitoring of blood pressure and timely management in patients who receive thrombolysis. Therefore, maintaining strict blood pressure control is essential to mitigate this risk and reduce the likelihood of further bleeding episodes. Additionally, regular assessment of neurological status and monitoring for signs of worsening hemorrhagic complications are essential components of post‐thrombolysis care.

## Author Contributions

**Abdallah Alwali:** conceptualization, resources, writing – original draft, writing – review and editing. **Mohanad Faisal:** conceptualization, resources, writing – review and editing. **Naveed Akhtar:** supervision.

## Consent

Written informed consent was obtained from the patient's family, as the patient had a low GCS.

## Conflicts of Interest

The authors declare no conflicts of interest.

## Data Availability

Data sharing not applicable to this article as no datasets were generated or analysed during the current study.
